# Lower Respiratory Tract Pathogens and Their Antimicrobial Susceptibility Pattern: A 5-Year Study

**DOI:** 10.3390/antibiotics10070851

**Published:** 2021-07-13

**Authors:** Biagio Santella, Enrica Serretiello, Anna De Filippis, Folliero Veronica, Domenico Iervolino, Federica Dell’Annunziata, Roberta Manente, Francesco Valitutti, Emanuela Santoro, Pasquale Pagliano, Massimiliano Galdiero, Giovanni Boccia, Gianluigi Franci

**Affiliations:** 1 Section of Microbiology and Virology, University Hospital “Luigi Vanvitelli”, 80138 Naples, Italy; bi.santella@gmail.com (B.S.); enrica.serretiello@unicampania.it (E.S.); veronica.folliero@unicampania.it (F.V.); roberta.manente@studenti.unicampania.it (R.M.); massimiliano.galdiero@unicampania.it (M.G.); 2 Department of Experimental Medicine, University of Campania “Luigi Vanvitelli”, 80138 Naples, Italy; anna.defilippis@unicampania.it (A.D.F.); federica.dellannunziata@unicampania.it (F.D.); 3 Department of Public Health and Infectious Diseases, Sapienza University of Rome, 00185 Rome, Italy; iervolino.1886704@studenti.uniroma1.it; 4 Clinical Pediatrics and Pediatrics, University Hospital “San Giovanni di Dio e Ruggi d’Aragona”, 84131 Salerno, Italy; francesco.valitutti@gmail.com; 5 Department of Medicine, Surgery and Dentistry “Scuola Medica Salernitana”, University of Salerno, 84081 Baronissi, Italy; esantoro@unisa.it (E.S.); ppagliano@unisa.it (P.P.); 6 Dai Dipartimento di Igiene Sanitaria e Medicina Valutativa U.O.C. Patologia Clinica E Microbiologica, Azienda Ospedaliero-Universitaria S. Giovanni di Dio e Ruggi D’Aragona Scuola Medica Salernitana, Largo Città di Ippocrate, 84131 Salerno, Italy

**Keywords:** lower respiratory tract infections, antimicrobial resistance, epidemiology, nosocomial infections, antimicrobial stewardship

## Abstract

Lower respiratory tract infections (LRTIs) are the most common infections in humans. It is estimated that 2.74 million deaths worldwide occur each year due to LRTIs. The aim of the study was to determine the frequency and antibiotic susceptibility pattern of microorganisms isolated from respiratory samples of patients with LRTIs. Between January 2015 and December 2019, a total of 7038 sputum and bronchoaspirate samples from suspected LRTI patients were collected. Among them, 2753 samples (39.1%) showed significant microbial growth on culture media. The LRTI rate was higher in patients with male gender (67.1%) and with age between 40–59 years (48.6%). The microorganism identification and antibiotic susceptibility testing were performed with Vitek 2. Out of 4278 isolates species, 3102 (72.5%) were Gram-negative bacteria, 1048 (24.5%) were Gram-positive bacteria, and 128 (3.0%) were *Candida* spp. Major microorganisms isolated were *Acinetobacter baumannii* (18.6%), *Staphylococcus aureus* (15.2%), *Pseudomonas aeruginosa* (14.2%), and *Klebsiella pneumoniae* (10.9%). In antimicrobial susceptibility testing, *Staphylococcus aureus* isolates were mostly resistant to Penicillin G (84.1%) and Oxacillin (48.1%), whereas they demonstrated maximum sensitivity to Tigecycline (100%) and Linezolid (99.5%). Among Gram-negative isolates, *Acinetobacter baumannii* showed maximum sensitivity to Colistin but was resistant to other antibiotics (95–99%). *Klebsiella pneumoniae* isolates were mostly resistant to Cefotaxime (72.7%) and sensitive to Gentamicin (54.3%), and *Pseudomonas aeruginosa* was resistant to Ciprofloxacin (40.3%) and sensitive to Amikacin (85.9%). Gram-negative bacteria represented the species most commonly isolated. A high rate of antimicrobial resistance was observed in this study. In conclusion, the correct identification of causative microorganisms and their susceptibility patterns to antibiotics is crucial for choosing targeted and effective antibiotic therapy in LRTIs, and to prevent the emergence of multidrug-resistant bacteria.

## 1. Introduction

Lower respiratory tract infections (LRTIs) are the most common infections in humans. It is estimated that 2.74 million deaths worldwide occur each year due to LRTIs [1]. The commonest LRTIs are acute bronchitis, acute trachea bronchitis, chronic bronchitis, and pneumonia, which account for 4.4% of all hospital admissions and are associated with high morbidity, mortality, and excessive health costs [2,3,4]. The incidence and related mortality due to LRTIs can be influenced by several factors, including age, gender, season, the type of population at risk, but mainly to antibiotic therapy, the distribution of causative agents, and the prevalence of antimicrobial resistance [5,6]. The microbial aetiology of LRTIs and their susceptibility profile to antibiotics varies in different geographic regions [7,8]. The most common bacterial agents of LRTIs are Gram-positive bacteria such as *Staphylococcus aureus* and *Enterococcus* spp., and Gram-negative bacteria such as *Pseudomonas* spp., *Acinetobacter* spp., *Klebsiella pneumoniae,* and *Haemophilus influenzae* [9,10,11,12].

Due to the severity of these infections, there is an urgent need to adopt empirical antimicrobial treatment, before receiving the result on bacterial aetiology and antimicrobial susceptibility patterns [13,14]. Unfortunately, the ongoing spread of extended-spectrum β-lactamases and carbapenems has begun to limit the clinical effectiveness of β-lactam agents. This trend is presumably due to the empirical administration of antibacterial therapy [15,16]. The situation is further complicated by the emergence of multi-resistant pathogens, such as *Klebsiella pneumoniae* carbapenemase and *Haemophilus influenzae* 𝛽-lactamase [17,18]. Therefore, current knowledge of bacterial etiology and their antimicrobial susceptibility pattern would help to choose the antimicrobial therapy for bacterial LRTIs, to limit the development of antimicrobial resistance and reduce overall management costs [19,20,21]. The aim of this study was to describe the prevalence and patterns of antimicrobial sensitivity of microorganisms isolated from respiratory samples of patients with LRTIs, admitted to the San Giovanni di Dio e Ruggi d’Aragona Hospital (Salerno, Italy), to improve treatment protocols.

## 2. Results

### 2.1. Incidence of LTRIs in Studied Patients

From 2015 to 2019, a total of 7038 samples of sputum (*n* = 3113) and bronchoaspirate (*n* = 3925) were processed according to the standard microbiological methods. The LRTIs were confirmed by microscopic examination, with more than 25 leukocytes per field and with the presence of microorganisms. Out of them, 2753 samples (39.1%) produced a significant growth of microorganisms on culture media (Table 1).

**Figure 1:**
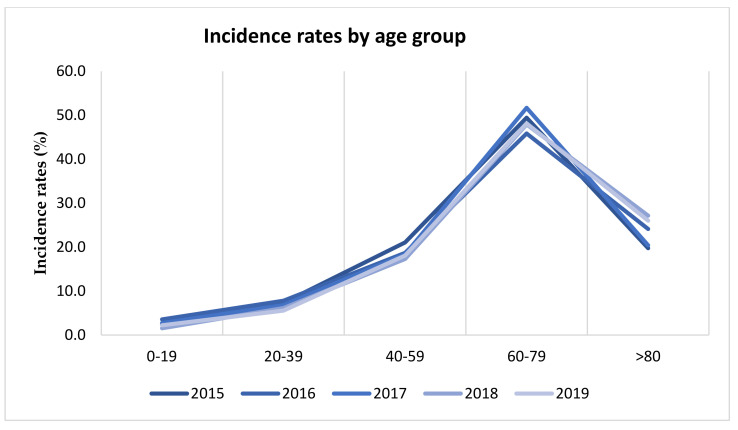
Distribution of positive cases by age group.

The average incidence of positive bronchoaspirate was 50.34%, double that of positive sputum 24.96% (<0.01). About 2.5% of patients were in the age group of fewer than 19 years, 6.7% in 20–39 years, 18.7% in 40–59 years, 48.6% in 60–79 years and 23.5% were of more than 80 years of age (Figure 1). Regarding gender, the LRTIs rate positive was higher in males than in females, 67.1% (1922) and 32.9% (831), respectively (>0.01).

### 2.2. Isolated Bacteria

All pathogens identified over the five years of study, with respective incidence rates, were provided as additional data (Supplementary Table S1). Out of 2753 samples positive, 4278 species have been isolated and analyzed (Table 2).

The rates of the number of isolates per positive sample are shown in Table 3, in 74.7% of cases there was monomicrobial growth, while in 25.1% there was significant polymicrobial growth. In the last year, only 15.2% of positive samples showed the growth of two microorganisms (Table 3).

Out of 4278 isolates identified, 3102 (72.5%) were Gram-negative bacteria, 1048 (24.5%) were Gram-positive bacteria, and 128 (3.0%) were *Candida* spp. (Table 4).

**Figure 2:**
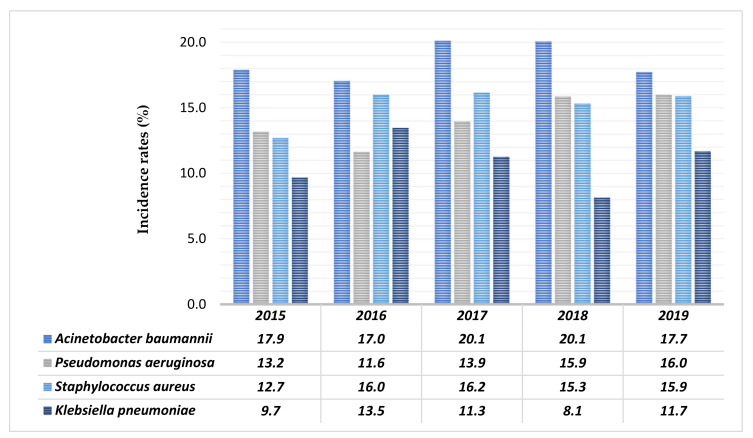
Annual incidence rates of the main bacteria isolated from positive samples.

Figure 2 shows the distribution of main microorganisms isolated from the LRTIs samples of hospitalized patients. The most common bacteria isolated were *Acinetobacter baumannii, Pseudomonas aeruginosa, Staphylococcus aureus*, and *Klebsiella pneumoniae*.

### 2.3. Prevalence of Antimicrobial Resistance among LRTIs Bacteria

A very high rate of resistance (98–100%) was observed among *Acinetobacter baumannii* isolates to Amoxicillin/Clavulanic acid, Cefotaxime, Ciprofloxacin, Ertapenem, Gentamicin, Imipenem, and Trimethoprim/Sulfamethoxazole. Only for Colistin, the isolate showed the maximum sensitivity with a rate of resistance less than 2% (Table 5).

Among the Gram-negative, *Pseudomonas aeruginosa* was the second most frequently isolated species from positive samples. Against *P. aeruginosa*, Colistin was the drug that showed the lowest resistance rates, less than 9%, despite the increasing trend during the study years, going from 3.8% in 2015 to 5.7% in 2019. However, Gentamicin showed a negative trend, with a decrease in resistance rate from 36.1% in 2015 to 12.3% in 2019. A similar trend was shown for Amikacin with a decrease in resistance rate, less than ten percentage points in the last year (from 15.5% to 4.9%). Among the Carbapenems class, Meropenem showed lower resistance rates compared to Imipenem, with a difference of around ten percentage points. Furthermore, Imipenem shows a negative trend in resistance rates over the years, going from 44.9% in 2015 to 33.3% in 2019. Ciprofloxacin showed the highest resistance rates compared to the others. Ceftazidime, in the Cephalosporins class, showed high resistance rates in 2015 (44.4%), higher than Cefepime (34.3%), but in the last year, the resistance rate was less to Cefepime (22.1% vs. 25.0%), due to negative trends. Moreover, Ciprofloxacin showed higher resistance rates than the other antibiotics described (Table 6).

Among the Gram-positive bacteria isolated, the most frequent species was *Staphylococcus aureus*. Resistance rates of *S. aureus* to Penicillin G were above 83% in 2016–2019, higher than in 2015. Moreover, to Oxacillin, *S. aureus* showed an increasing resistance trend from 2015 to 2019, going from 32.4% to 60.7%. Similar results were showed for the Macrolide class, in which Erythromycin showed higher resistance rates in 2019 (51.9%). This antibiotic class, showed a similar trend, with a higher resistance rate in 2017. Moreover, Clindamycin has observed a positive trend, with an increase in the resistance rate, more than ten percentage points, from 2015 to 2019. The rate of resistance to Levofloxacin, showed a positive trend, with different resistance rates, and an increase to more than twenty percentual points in confront to the first year of study, from 28.1% in 2015 to 54.9% in 2019. The Aminoglycosides and Tetracyclines class showed resistance rates much lower than the previously listed classes, showing maximum sensibility to Tigecycline, with resistance rates to 0%. Glycopeptides class showed similar results, in which, Vancomycin and Teicoplanin showed very low resistance rates, but a worrying increasing trend over the years of study, going from 0% in 2015 to 4.2% and 2.5% in 2019, respectively. Finally, for Linezolid, resistance rates better than the previous ones have been recorded, with a percentage of less than 2.0% from 2015 to 2019 (Table 7).

*Klebsiella pneumoniae* was the third most frequently isolated Gram-negative species, with a mean percentage of 10.9%. Against *K. pneumoniae*, the tested antibiotics belonging to the Penicillin and Cephalosporin class showed similar high rates of resistance, exceeding 70%, including Ciprofloxacin. However, Amoxicillin-clavulanate and Cefepime have shown decreasing resistance rates over the years, with a difference of more than twenty percentage points over the past year. Moreover, the Carbapenem class showed high resistance rates with the highest values exceeding 70%. Resistance values lower than those discussed previously, around 50%, are highlighted by the class of Aminoglycosides and Tetracyclines. Finally, Colistin showed the lowest resistance rates compared to the other antibiotics, around 15%, ranking it as the most efficient antibiotic against this species (Table 8).

## 3. Discussion

The aim of the study was to determine the prevalence of microorganisms responsible to LRTIs in our hospital and their susceptibility profile to antibiotics. From 2015 to 2019, 7038 sputum and bronchoaspirate samples were analyzed, among them 2753 (39.1%) produced a significant growth of microorganisms on culture media, with a higher incidence in bronchoaspirate samples compared to sputum (52.7% vs. 25.1%). Recently published articles show similar incidence rates to ours [22,23,24].

LRTIs were more common in males than females (67.1% vs. 32.9%). Male prevalence of LRTI may be due to some associated risk factors for respiratory tract infection such as smoking, alcohol consumption and COPD [12,25]. Age range distribution showed that the incidence of LTRIs increases rapidly with increasing age, with a maximum incidence between 60 and 79 years. Among 4278 pathogenic microorganism isolates, 72.5% were Gram-negative bacteria, 24.5% were Gram-positive bacteria, and 3.0% were *Candida* spp. In similar studies, Gram-negative bacteria represented the species most commonly isolated from samples by patients of lower respiratory tract infections [26,27]. In the current investigation, monomicrobial growth was found in 74.7% of the cases, whereas 25.3% were polymicrobial, and identification of the polymicrobial infection is very important for treatment strategies. In another study, monomicrobial growth was found in 80% of cases, whereas 20% were polymicrobial [28]. The major microorganisms causing LRTI were *Acinetobacter baumannii*, *Staphylococcus aureus*, *Pseudomonas aeruginosa*, and *Klebsiella pneumoniae*. This observation is similar to other studies, such that of Christopher Aye Egbe et al., in which the most frequent mixed infection was caused by *Klebsiella* spp. and *Pseudomonas* spp. [12,24,29].

Antimicrobial susceptibility test performed on *Acinetobacter baumannii* isolates showed that Colistin was the most effective (nearly 100% sensitivity) and Amoxicillin/Clavulanic acid, Cefotaxime, Oxacillin, Ciprofloxacin, Ertapenem, Gentamicin, Imipenem, and Trimethoprim/Sulfamethoxazole were the least effective ones (98–100% resistance). The causes may be due to the high propensity of this species to easily acquire resistance genes and the ability to persist and multiply in a hospital setting. The high rates of resistance to first-line antimicrobial drugs shown by *A. baumannii* isolate highlight the need to find new effective molecules to counter this threat [30,31,32,33,34].

*Pseudomonas aeruginosa* was the second commonest organism among Gram-negative bacteria isolated. In the case of *P. aeruginosa*, Gentamycin, Amikacin, and Colistin were the most effective antibiotics (less to 20% resistance), contrary to Piperacillin/Tazobactam and Ciprofloxacin that was the least effective (40–50% resistance). The antibiotic resistance rates shown in *P. aeruginosa* are similar to results shown by a study by Yayan J. et al. [35]

The most species isolated of Gram-positive was *Staphylococcus aureus*. This species was found to be mostly resistant against Penicillin G and medium resistant to Oxacillin, followed by Macrolides, Lincosamides and Fluoroquinolones class. The antibiotics more efficient against *S. aureus* were Gentamicin, Tetracycline, Tigecycline, Teicoplanin, Vancomycin, and Linezolid, with a rate of resistance less to 10%. Oxacillin, Vancomycin, and Teicoplanin showed very low resistance rates, but a worrying increasing trend over the years of study. This event may have been caused by the increase in Oxacillin-resistant *S. aureus* MRSA, which are treated with Vancomycin and Teicoplanin; consequently, an increase in resistance to the latter has been observed. The same event has been documented in other articles [36,37,38,39,40].

Among Gram-negative bacteria, in terms of frequency, *P. aeruginosa* was followed by *Klebsiella pneumoniae*. This species was mostly resistant to Penicillin, Cephalosporins, and Fluoroquinolones class (above 70%). Moreover, in this case, the most efficient antibiotic was Colistin, with resistance rates higher than 10%, while the Carbapenems, Aminoglycosides and Tetracyclines class have higher resistances, around 45%. In a study by Ahmed et al., *K. pneumoniae* showed similar resistance rates, except for Piperacillin/Tazobactam, with a reported resistance rate of 18.2%, lower than that shown in our study (73.3%) [41]. This difference could be due to a different empirical antimicrobial treatment and a different geographic region, as described by Prestinaci et al. [42]. Very recent reports indicated Gram-negative bacteria were the major microorganisms involved in LRTIs [12,43] and were reported to increase resistance to Carbapenems and Fluoroquinolones for *K. pneumoniae*. Another hand, among Gram-positive, *S. aureus* was the main bacteria isolated reported and showed high susceptibility to Vancomycin and Linezolid, findings are the same as observed in our study. According to a recent WHO report on the epidemiology of infectious diseases, LRTI tops the list in developing countries [44]. This study reveals the incidence of the main pathogens responsible for LRTI and their resistance to the most commonly used antibiotics in hospital settings [45,46]. The results show high rates of resistance by the most commonly isolated bacteria. Resistance to Fluoroquinolones and 3rd generation Cephalosporins is rapidly emerging [47]. This may be due to irrational drug abuse and the resulting mutation of pathogenic microorganisms [48,49].

## 4. Materials and Methods

### 4.1. Samples Collection

The present retrospective study was conducted in the Microbiology department of University Hospital “San Giovanni di Dio e Ruggi d’Aragona” in the period between January 2015 and December 2019.

Sputum and bronchial aspiration samples from patients with suspected lower respiratory tract infection (LRTI) were analyzed. A total of 7038 samples were collected from patients of all age and gender groups.

### 4.2. Identification and Antimicrobial Susceptibility Testing

The samples were collected in sterile containers and immediately transported to the bacteriology laboratory and were processed further. Only those samples with an adequate amount of sputum were accepted. Those that contained an inadequate amount of sputum for analysis or that contained only saliva were excluded.

Sputum specimen for bacteriological culture was subjected to Gram-staining and examined microscopically. In microscopic examination sputum smear containing less than 10 squamous epithelial cells and more than 25 leucocytes or pus cells per low power, field confirmed the reliability of the specimen, indicating that it was not contaminated with saliva. The samples of sputum which were very thick and mucoid were first homogenized with commercially available sputasol containing 0.01% dithiothreitol and were incubated at 37 °C for 30 min for complete homogenization of sputum.

All samples were immediately plated on Chocolate agar, blood agar, MacConkey, and Sabouraud Glucose agar medium (Oxoid, Hampshire, UK), and were incubated at 37 °C. The Chocolate agar was incubated at 37 °C in a 5% CO_2_ atmosphere. After 18–36 h of incubation, each plate was examined, and bacterial identification and antimicrobial susceptibility test were performed.

Sputum or bronchoaspirate showing less than 10^4^ CFU/ml by semiquantitative culture were regarded as commensal or contaminant and were excluded. The bacterial identification and antimicrobial susceptibility test were performed utilizing a Vitek 2 (bioMerieux, Marcy l’Etoile, France), using an identification card (ID-GN for Gram-Negative, ID-GP for Gram-positive, YST for yeast) and the AST-659 (for Staphylococci), AST-658 (for Enterococci and *S. agalactiae*), AST-ST03 (for Pneumococci), AST-379 (for *Enterobacteriaceae*), and AST-397 (for GN non-fermenters) susceptibility cards, according to the manufacturer’s instructions. The results of antimicrobial susceptibility were interpreted as “susceptible”, “resistant”, or “intermediate” according to EUCAST guidelines. The Quality Control process encompasses the annual service and certification of the instrument by bioMérieux and the Quality Control of each lot of Gram-negative (GN), Gram-positive (GP) cards using four strains: *Enterococcus* ATCC 700,327 and *Staphylococcus aureus* ATCC 29,213 for GP; *Enterobacter* ATCC 700,323 and *Klebsiella oxytoca* ATCC 700,324 for GN.

The following antibiotics were included in the present study: amikacin (AMK), amoxicillin/clavulanic acid (AMC), azithromycin (AZM), cefepime (FEP), cefotaxime (CTX), ceftazidime (CAZ), ciprofloxacin (CIP), clarithromycin (CLR), colistin (COL), ertapenem (ETP), erythromycin (ERY), gentamicin (GEN), imipenem (IPM), levofloxacin (LVX), linezolid (LNZ), meropenem (MEM), moxifloxacin (MFX), oxacillin (OXA), piperacillin/tazobactam (TZP), teicoplanin (TEC), tigecycline (TCG), vancomycin (VAN).

### 4.3. Ethical Consideration Statement

Ethical approval by the Human Research Ethics Committee was not requested. The present study used laboratory management data, collected from a database. This is a retrospective study and not directly associated with patients.

### 4.4. Statistical Analysis

Demographic data of patients, including age, gender, isolated strain(s), and drug sensitivity results, were used for the analysis. The crude incidence and age- and sex-standardized incidence were calculated. The chi-framework test was used to compare the differences in the incidence of bacteria in hospitalized patients and the differences among antibiotic sensitivities over the range of years considered in the study. A chi-square test was used to verify the possible associations between the categorical variables, while the Cochran–Armitage trend test was used to verify the existence of a trend, the existence of a trend was checked only for antibiotics that showed statistically significant differences in the distribution of resistance during the years under consideration, an alpha equal to 5% was considered for both tests, therefore those associations that had a *p*-value < 0.05 were considered statistically significant. The IBM Statistical Package for Social Sciences Version 22.00 (SPSS Inc., Chicago, IL, USA) was used for data analysis.

## 5. Conclusions

Bacterial aetiology varies in different regions and populations. Therefore, infection surveillance studies have become increasingly necessary and important to control LRTIs. The correct identification of pathogenic microorganisms and their susceptibility patterns to antibiotics can be useful for our healthcare professionals to choose the most targeted and effective antibiotic therapy. Furthermore, this study could represent an alarm for the competent authorities to develop effective policies on the precise and rational prescription of antibiotics. However, our research has certain limitations, partly because it was only focused on LRTI patients admitted to the San Giovanni di Dio e Ruggi d’Aragona Hospital. Therefore, multicenter, longitudinal, prospective research is required to confirm our findings. However, molecular diagnostic tests will need to be strengthened for faster results. All this could reduce the phenomenon of antibiotic resistance and ensure maximum safety for the health of patients.

## Figures and Tables

**Table 1:** Cases of LTRIs distributed by year.

*Samples*	2015	2016	2017	2018	2019	Total
*Positive bronchoaspirates*	404	409	341	402	420	1976
*Total bronchoaspirates*	597	617	752	939	1020	3925
*Positive sputum*	171	128	112	154	212	777
*Total sputum*	628	492	527	688	778	3113

**Table 2:** The number of species isolated per type of positive sample.

Number Isolated	2015	2016	2017	2018	2019	Total
Broncoaspirate	605	614	599	661	655	3134
Sputum	249	194	198	228	275	1144
Total	854	808	797	889	930	4278

**Table 3:** The rates of the number of isolates per positive sample.

*Number of Isolated Species per Sample*	*1*	*2*	*3*
***2015***	69.0	24.9	5.6
***2016***	71.3	24.0	4.4
***2017***	74.2	21.9	3.5
***2018***	76.0	21.5	2.5
***2019***	82.9	15.2	1.8

**Table 4:** Subdivision of microorganisms isolated from positive samples expressed in percentage.

Microorganisms	2015	2016	2017	2018	2019
**Gram positive**	23.3	25.5	25.1	23.7	25.0
**Gram negative**	73.5	71.7	72.0	73.5	71.8
**Fungi**	3.2	2.8	2.9	2.7	3.2

**Table 5:** Antimicrobial resistance profile of *Acinetobacter baumannii* to antibiotics used in Hospital.

***Acinetobacter baumannii***								
**Drug Class**	**Antibiotics**	**2015**	**2016**	**2017**	**2018**	**2019**	*	**
**Penicillins**	Amoxicillin-clavulanate	100 (148)	100 (129)	100 (127)	100 (148)	100 (135)	-	-
**Cephalosporins**	Cefotaxime	100 (148)	100 (129)	100 (127)	100 (148)	100 (135)	-	-
**Fluoroquinolone**	Ciprofloxacin	98.0 (148)	95.3 (129)	94.5 (127)	95.3 (148)	94.1 (135)	0.55	-
**Carbapenems**	Ertapenem	100 (148)	100 (129)	100 (127)	100 (148)	100 (135)	-	-
	Imipenem	97.3 (148)	94.6 (129)	94.5 (127)	94.2 (148)	96.3 (135)	0.68	-
**Aminoglycoside and Tetracycline**	Gentamicin	94.6 (148)	94.6 (129)	85.8 (127)	89.2 (148)	94.8 (135)	0.02	0.46
**DHFR inhibitors**	Trimethoprim/Sulfam.	97.3 (148)	94.6 (129)	94.5 (127)	94.2 (148)	96.3 (135)	0.41	-
**Polypeptide**	Colistin	0 (148)	1.6 (129)	0 (127)	0 (148)	1.6 (135)	0.16	-

* *p*-value with chi-square, ** *p*-value with Cochran–Armitage trend test.

**Table 6:** Antimicrobial resistance profile of *Pseudomonas aeruginosa* to antibiotics used in Hospital.

***Pseudomonas aeruginosa***								
**Drug Class**	**Antibiotics**	**2015**	**2016**	**2017**	**2018**	**2019**	*	**
**Penicillins**	Piperacillin/Tazobactam	47.2 (106)	40.0 (85)	28.0 (50)	33.9 (112)	33.9 (121)	0.10	-
**Cephalosporins**	Ceftazidime	44.4 (108)	39.8 (88)	51.2 (86)	27.0 (115)	22.1 (122)	<0.01	<0.01
	Cefepime	34.3 (108)	28.4 (88)	37.1 (89)	23.3 (90)	25.0 (120)	0.16	-
**Fluoroquinolones**	Ciprofloxacin	51.8 (108)	35.2 (88)	45.3 (86)	27.6 (116)	41.8 (122)	0.03	0.05
**Carbapenems**	Imipenem	44.9 (107)	47.7 (88)	39.5 (86)	39.3 (89)	33.3 (120)	0.25	-
	Meropenem	27.8 (108)	31.8 (88)	28.2 (85)	20.9 (115)	23.1 (121)	0.40	-
**Aminoglycosides and Tetracycline**	Gentamicin	36.1 (108)	25.0 (88)	37.2 (86)	10.3 (116)	12.3 (122)	<0.01	<0.01
	Amikacin	15.5 (103)	18.4 (87)	24.7 (85)	6.9 (116)	4.9 (122)	<0.01	<0.01
**Polypeptide**	Colistin	3.8 (104)	2.5 (81)	0 (63)	8.3 (84)	5.7 (87)	0.12	-

* *p*-value with chi-square, ** *p*-value with Cochran–Armitage trend test.

**Table 7:** Antimicrobial resistance profile of *Staphylococcus aureus* to antibiotics used in Hospital.

***Staphylococcus aureus***								
**Drug Class**	**Antibiotics**	**2015**	**2016**	**2017**	**2018**	**2019**	*	**
**Penicillins**	Penicillin G	78.1 (105)	83.5 (121)	89.3 (103)	86.1 (101)	83.3 (120)	0.12	-
	Oxacillin	32.4 (105)	35.5 (121)	54.4 (103)	56.4 (101)	60.7 (112)	<0.01	<0.01
**Macrolides**	Erythromycin	40.0 (105)	46.3 (121)	59.7 (103)	45.0 (100)	51.8 (112)	0.06	-
	Azithromycin	38.5 (96)	45.1 (102)	59.2 (103)	44.3 (70)	NA	0.03	0.10
	Clarithromycin	38.5 (96)	45.1 (102)	59.2 (103)	44.3 (70)	NA	0.03	0.10
**Lincosamides**	Clindamycin	36.3 (102)	40.7 (118)	52.4 (103)	40.7 (113)	50.0 (112)	0.09	-
**Fluoroquinolones**	Levofloxacin	28.1 (96)	40.2 (102)	58.3 (103)	45.5 (101)	54.9 (113)	<0.01	<0.01
**Aminoglycosides and Tetracycline**	Gentamicin	8.6 (105)	12.4 (121)	2.9 (103)	4.0 (101)	5.4 (112)	0.03	0.05
	Tetracycline	8.6 (105)	2.5 (121)	2.9 (103)	4.0 (101)	5.8 (120)	0.17	-
	Tigecycline	0 (96)	0 (113)	0 (103)	0 (113)	0 (121)	-	-
**Glycopeptides**	Teicoplanin	0 (105)	0 (102)	1.0 (103)	0 (113)	4.2 (118)	<0.01	<0.01
	Vancomycin	0 (96)	0 (121)	1.0 (103)	0 (113)	2.5 (120)	0.10	-
**Oxazolidinones**	Linezolid	0 (105)	1.7 (121)	0 (103)	0 (113)	0.8 (121)	0.31	-

* *p*-value with chi-square, ** *p*-value with Cochran–Armitage trend test.

**Table 8:** Antimicrobial resistance profile of *Klebsiella pneumoniae* to antibiotics used in hospital.

***Klebsiella pneumoniae***								
**Drug Class**	**Antibiotics**	**2015**	**2016**	**2017**	**2018**	**2019**	*	**
**Penicillins**	Amoxicillin-clavulanate	78.3 (83)	75.8 (91)	57.8 (71)	57.6 (59)	59.3 (81)	<0.01	<0.01
	Piperacillin/Tazobactam	77.6 (85)	80.4 (102)	66.2 (71)	65.0 (60)	77.5 (89)	0.09	-
**Cephalosporins**	Cefepime	74.2 (85)	77.4 (102)	57.7 (71)	57.7 (59)	44.9 (91)	<0.01	0.01
	Cefotaxime	81.2 (80)	77.4 (102)	66.2 (71)	56.7 (60)	82.0 (89)	<0.01	0.10
	Ceftazidime	75.3 (85)	78.4 (102)	66.2 (71)	57.6 (59)	82.0 (89)	<0.01	<0.03
**Fluoroquinolones**	Ciprofloxacin	76.5 (85)	77.4 (102)	64.8 (71)	53.3 (60)	82.0 (89)	<0.01	0.24
**Carbapenems**	Ertapenem	57.6 (59)	72.5 (102)	47.9 (71)	46.7 (60)	70.8 (89)	<0.01	<0.01
	Meropenem	70.2 (84)	72.5 (102)	48.6 (70)	45.8 (59)	71.6 (88)	<0.01	0.23
**Aminoglycosides and Tetracycline**	Gentamicin	45.9 (85)	49.0 (102)	52.1 (71)	36.7 (60)	44.9 (89)	0.47	0.50
	Tigecycline	53.1 (64)	64.4 (101)	52.1 (71)	44.1 (59)	59.1 (88)	0.12	-
**DHFR inhibitors**	Trimethoprim/Sulfam.	74.1 (85)	72.5 (102)	63.4 (71)	41.7 (60)	34.8 (89)	<0.01	<0.01
**Polypeptide**	Colistin	33.7 (83)	28.3 (99)	11.1 (54)	2.3 (39)	42.5 (73)	<0.01	<0.01

* *p*-value with chi-square, ** *p*-value with Cochran–Armitage trend test.

## Data Availability

Epidemiological data used to support the results of this study are included in the article.

## Supplementary Materials

The following are available online at https://www.mdpi.com/article/10.3390/antibiotics10070851/s1, Table S1: Percentage trend of all bacterial isolates that caused LTRIs infections.
