# Supplementary material for: Lower Respiratory Tract Pathogens and Their Antimicrobial Susceptibility Pattern: A 5-Year Study
Source: Antibiotics (Basel). 2021 Jul 13;10(7):851. doi: 10.3390/antibiotics10070851 (PMC8300710; doi:10.3390/antibiotics10070851)
Supplement: Supplementary file 1 [file antibiotics-10-00851-s001.zip › antibiotics-1250230-supplementary.pdf]

Table S1: Percentage trend of all bacterial isolates that caused LTRIs infections.

Isolated species	2015	%	2016	%	2017	%	2018	%	2019	%
Acinetobacter baumannii	148	17.9	129	17.0	127	20.1	148	20.1	135	17.7
Pseudomonas aeruginosa	109	13.2	88	11.6	88	13.9	117	15.9	122	16.0
Staphylococcus aureus	105	12.7	121	16.0	102	16.2	113	15.3	121	15.9
Klebsiella pneumoniae	80	9.7	102	13.5	71	11.3	60	8.1	89	11.7
Escherichia coli	43	5.2	59	7.8	62	9.8	72	9.8	64	8.4
Stenotrophomonas maltophilia	24	2.9	21	2.8	23	3.6	31	4.2	33	4.3
Providencia stuartii	22	2.7	17	2.2	25	4.0	17	2.3	6	0.8
Proteus mirabilis	20	2.4	29	3.8	28	4.4	35	4.7	29	3.8
Enterobacter aerogenes	64	7.7	18	2.4	9	1.4	7	0.9	7	0.9
Candida spp	52	6.3	25	3.3	5	0.8	11	1.5	11	1.4
Serratia marcescens	18	2.2	12	1.6	4	0.6	11	1.5	13	1.7
Streptococcus pneumoniae	15	1.8	12	1.6	6	1.0	7	0.9	14	1.8
Enterobacter cloacae	13	1.6	19	2.5	17	2.7	18	2.4	19	2.5
Enterococcus faecalis	12	1.4	14	1.8	9	1.4	21	2.8	21	2.8
Haemophilus influenzae	12	1.4	9	1.2	7	1.1	10	1.4	11	1.4
Klebsiella oxytoca	10	1.2	15	2.0	7	1.1	18	2.4	13	1.7
Staphylococcus haemolyticus	10	1.2	12	1.6	5	0.8	9	1.2	11	1.4
Citrobacter koseri	7	0.8	7	0.9	4	0.6	3	0.4	11	1.4
Staphylococcus epidermidis	7	0.8	8	1.1	1	0.2	5	0.7	3	0.4
Acinetobacter junii	5	0.6	4	0.5	2	0.3	1	0.1	1	0.1
Citrobacter freundii	5	0.6	8	1.1	2	0.3	5	0.7	7	0.9
Raoultella planticola	5	0.6	1	0.1	2	0.3	2	0.3	0	0.0
Sphingomonas paucimobilis	5	0.6	0	0.0	0	0.0	1	0.1	0	0.0
Streptococcus agalactiae	5	0.6	4	0.5	7	1.1	1	0.1	1	0.1
Enterococcus faecium	4	0.5	3	0.4	2	0.3	4	0.5	5	0.7
Morganella morganii	4	0.5	4	0.5	4	0.6	1	0.1	3	0.4
Streptococcus viridans group	4	0.5	3	0.4	0	0.0	0	0.0	0	0.0
Achromobacter denitrificans	3	0.4	2	0.3	3	0.5	1	0.1	1	0.1
Pseudomonas fluorescens	3	0.4	3	0.4	1	0.2	3	0.4	3	0.4
Pseudomonas putida	3	0.4	3	0.4	3	0.5	1	0.1	4	0.5
Achromobacter xylosoxidans	2	0.2	0	0.0	2	0.3	2	0.3	1	0.1
Acinetobacter lwoffii	2	0.2	1	0.1	1	0.2	1	0.1	1	0.1
Raoultella ornithinolytica	2	0.2	1	0.1	0	0.0	0	0.0	0	0.0
Serratia liquefaciens	2	0.2	1	0.1	1	0.2	0	0.0	1	0.1
Serratia plymuthica	2	0.2	0	0.0	0	0.0	1	0.1	1	0.1
Kluyvera cryocrescens	1	0.1	2	0.3	1	0.2	1	0.1	0	0.0
Total	**828**		**757**		**631**		**738**		**762**	
